# The cost bloodstream infections caused by antimicrobial susceptible and non-susceptible Enterobacteriaceae and *Staphylococcus aureus* in European hospitals

**DOI:** 10.1186/2047-2994-4-S1-O55

**Published:** 2015-06-16

**Authors:** AJ Stewardson, N Graves, A Allignol, S Harbarth

**Affiliations:** 1 University of Geneva Hospitals, Geneva, Switzerland; 2 Queensland University of Technology, Brisbane, Queensland, Australia; 3 Ulm University, Ulm, Germany

## Introduction

Antimicrobial resistance (AMR) represents a significant global threat. It is useful to estimate the economic burden of AMR as these represent potential cost savings from reducing the problem.

## Objectives

To determine the impact of antimicrobial resistance on cost attributable to bloodstream infections (BSIs) caused by *Staphylococcus aureus* and Enterobacteriaceae from a European hospital perspective.

## Methods

We performed a multicentre retrospective cohort study including acute inpatient episodes at ten European hospitals in 2010 and 2011. BSIs were the time-varying exposure of interest, with *S. aureus* classified as methicillin-susceptible (MSSA) or resistant (MRSA), and Enterobacteriaceae as third-generation-cephalosporin-susceptible (3GCSE) or resistant (3GCRE). We used multistate models to estimate excess length-of-stay (LOS). For each bacteria/susceptibility combination, we computed the attributable cost of a single BSI as the product of excess LOS and the bed-day value. We used two contrasting bed-day values: an economic value obtained by contingent valuation and the accounting cost derived by dividing the annual hospital budget by the number of bed-days. To estimate the annual hospital costs of each BSI, these BSI costs were multiplied by the expected number of BSI cases per year. We performed a probabilistic sensitivity analysis to account for parameter uncertainty.

## Results

Our cohort included 606,649 patients. Third-generation-cephalosporin-resistance significantly increased the hazard of death (1.5 [1.0–2.2]), excess LOS (4.9 [1.1–8.7]) and cost compared to susceptible strains, whereas methicillin resistance did not. Whilst 3GCSE BSI was associated with the lowest per-infection cost (€320 [95% credible interval, €80–€1,300] or €4,000 [€2,400–€6,700] using economic and accounting valuations, respectively), their relative frequency resulted in equal highest annual cost with MSSA (€77,000 [€19,000–€300,000] or €970,000 [€590,000–€1,600,000] using economic and accounting valuations, respectively).

## Conclusion

While BSI with *S. aureus* has a greater impact on mortality, excess LOS and cost than Enterobacteriaceae per infection, the impact of antimicrobial resistance is greater amongst BSIs caused by Enterobacteriaceae.

## Disclosure of interest

None declared.

